# Chitosan oligosaccharide suppresses osteosarcoma malignancy by inhibiting CEMIP via the PI3K/AKT/mTOR pathway

**DOI:** 10.1007/s12032-023-02165-9

**Published:** 2023-09-05

**Authors:** IlJin Sim, WonGyom Choe, JinJu Ri, Hang Su, Safwat Adel Abdo Moqbel, WeiQi Yan

**Affiliations:** 1 Department of Orthopedics, The Second Affiliated Hospital of Zhejiang University School of Medicine, Jiefang Campus, 88 Jiefang Road, Shangcheng District, Hangzhou, 310009 China; 2 Zhejiang University School of Medicine, Zhejiang University Huajiachi Campus, 268 Kaixuan Road, Jianggan District, Hangzhou, 310029 China; 3 The BioMed Innovation Institute of Hangzhou Medical College, Hangzhou, 310010 China; 4 Department of Emergency Medicine, The Second Affiliated Hospital of Zhejiang University School of Medicine, Jiefang Campus, 88 Jiefang Road, Shangcheng District, Hangzhou, 310009 China; 5 Clinical Institute, Pyongyang Medical University, Pyongyang, 999093 Democratic People’s Republic of Korea; 6 Department of Cardiology, Pyongyang Medical University Hospital, Pyongyang, 999093 Democratic People’s Republic of Korea

**Keywords:** Osteosarcoma, Chitosan oligosaccharide, CEMIP, PI3K, Pathway

## Abstract

Osteosarcoma is a malignant bone tumor that is prone to metastasize early and primarily affects children and adolescents. Cell migration-inducing protein (CEMIP) plays a crucial role in the progression and malignancy of various tumor diseases, including osteosarcoma. Chitosan oligosaccharide (COS), an oligomer isolated from chitin, has been found to have significant anti-tumor activity in various cancers. This study investigates the effects of COS on CEMIP expression in osteosarcoma and explores the underlying mechanism. In present study, in vitro experiments were conducted to confirm the inhibitory activity of COS on human osteosarcoma cells. Our results demonstrate that COS possesses inhibitory effects against human osteosarcoma cells and significantly suppresses CEMIP expression in vitro. Next, we studied the inhibition of the expression of CEMIP by COS and then performed bioinformatics analysis to explore the potential inhibitory mechanism of COS against signaling pathways involved in regulating CEMIP expression. Bioinformatics analysis predicted a close association between the PI3K signaling pathway and CEMIP expression and that the inhibitory effect of COS on CEMIP expression may be related to PI3K signaling pathway regulation. The results of this study show that COS treatment significantly inhibits CEMIP expression and the PI3K/AKT/mTOR signaling pathway, as observed both in vitro and in vivo. This study demonstrates that COS could inhibit the expression of CEMIP, which is closely related to osteosarcoma malignancy. This inhibitory effect may be attributed to the inhibition of the PI3K/AKT/mTOR signaling pathway in vitro and in vivo.

## Introduction

Osteosarcoma is the most common primary malignant bone tumor in children and adolescents. Long tubular bones, such as the femur and tibia, are the most common sites of osteosarcoma [1, 2]. Although treatment methods, including surgery and chemotherapy, have been established, and multiple clinical trials have been conducted to improve treatment outcomes, the overall survival rate remains unimproved due to the rapid growth rate and early metastasis of this tumor [2].

Cell migration-inducing protein (CEMIP or KIAA1199), located on chromosome 15q25.1, can be detected in the nucleus and the cytoplasm [3, 4]. It was first identified as a gene associated with hearing loss [5], but further research found that CEMIP is closely related to the progression and malignancy of various tumors, including pancreatic cancer and colorectal cancer [6, 7]. Studies have found that overexpression of CEMIP leads to resistance to cell immortalization and carcinogenesis in normal human cells and is also involved in cell death [8]. In breast and pancreatic cancers, CEMIP has been found to promote the proliferation, invasion, and metastasis of cancer cells [9, 10]. Moreover, increased expression of CEMIP has been found to promote the progression and metastasis of osteosarcoma and has been associated with poor prognosis of osteosarcoma [11]. Therefore, CEMIP has emerged as a potential therapeutic target to suppress tumor malignancy. However, research on the use of natural bioactive complexes to inhibit CEMIP expression is limited.

Chitosan oligosaccharide (COS) is a chitosan-derived oligomer with an average molecular weight < 10,000 kDa. Compared with chitosan, COS has a small molecular weight, better water solubility, and a higher absorption rate. Therefore, it has attracted considerable attention as a potential treatment for various diseases[12, 13]. In recent years, numerous studies have demonstrated significant anti-tumor activity of COS against various cancers [12, 14, 15]. COS exerts anti-tumor effects by direct inhibition of tumor cell activity and stimulation of the host immune system [16, 17].

The PI3K/AKT/mTOR pathway is one of the main pathways involved in regulating the expression of various target genes and is often mutated in tumor diseases. Abnormal activation of this pathway is closely related to various functional abnormalities and mechanisms of cells and tissues, including abnormal cell proliferation and apoptosis, tumor occurrence and progression, and drug resistance [18, 19]. Recent studies have demonstrated the association between the PI3K/AKT/mTOR signaling pathway and the regulation of CEMIP expression [20, 21]. These associations suggest that COS, a natural active anticancer compound shown to interfere with various signaling pathways, may have favorable effects on the expression of CEMIP. Therefore, the aim of the present study is to investigate the effects and underlying mechanisms of COS on CEMIP expression in osteosarcoma.

## Materials and methods

### Cell lines and culture

The two human osteosarcoma cell lines (MG63 and U2OS) and murine OS cell line (K7M2) used in the present study were purchased from the American type culture collection (ATCC).

The cells were cultured in Dulbecco's modified Eagle's medium (DMEM, Coring) containing 10% fetal bovine serum (Gibco), penicillin (100 IU/mL), and streptomycin (100 μg/mL NCM Biotech, Suzhou, China) at 37 °C with 5% CO_2_.

### Reagents

COS (DD = 90%, Mw = 3000 Da) was purchased from Zhejiang Golden Shell Pharmaceutical Co., Ltd. (Zhejiang, China). Dimethyl sulfoxide (DMSO, Sigma-Aldrich) and PBS were used as the negative control (NC) and the vehicle in this study. For flow cytometry assay, AnnexinV-FITC/PI Apoptosis kit was purchased from Multisciences (LIANKE, Zhejiang, China). BCA Protein Assay Kit, radioimmunoprecipitation assay (RIPA) buffer, protease inhibitor (PMSF), and Cell Counting Kit-8 (CCK8) were purchased from Beyotime (Shanghai, China).

### CCK-8 assay

CCK8 assay was carried out according to the manufacturer's protocol to confirm the cytotoxicity of COS on human osteosarcoma cells. MG63 and U2OS cells (5 × 10^3^/well) were seeded onto 96 well plates. After adhering to the wall, the cells were treated with different doses of COS for 24 h, 48 h, and 72 h. At indicated times, CCK8 solution (10 μL) was added to each well and incubated for 2 h at 37 °C. The absorbance at 450 nm and 620 nm was measured using a microplate reader (SpectraMax® ABS, Shanghai, China).

### Cell colony formation assay

Two human osteosarcoma cell lines were seeded and cultured in 6 well plates (1.5 × 10^3^ cells/well). After adhering to the wall, the cells were treated with various concentrations of COS (0, 7, 14, and 21 mg/mL) for 10 days. 4% polyformaldehyde (POM) was used to fix the colonies at room temperature. Then, the colonies were stained with 1% crystal violet solution for 20 min at room temperature. The colonies were observed and counted using a microscope (OLYMPUS, Japan).

### Wound healing assay

MG63 and U2OS cells were seeded into 6 well plates. After 24 h, a pipette tip (100 μL) was used to create a scratch across the cell layers to form a wound line. After washing 3 times with PBS, the cells were treated with different doses of COS (0, 7, 14 and 21 mg/mL) for 24 h. The cells were observed and photographed using a microscope, and the migration rates were analyzed using ImageJ 1.52.

### Transwell cell invasion assay

Transwell cell invasion assay was performed to analyze the effects of COS on the invasiveness of the osteosarcoma cells. The transwell chambers (8 μm filter) were pre-coated with 30 μL Matrigel (BD Bioscience, San Jose, CA, USA). The cells (5 × 10^4^/well) resuspended in 200 μL of serum-free DMEM medium were added to the upper chamber. The lower chambers were filled with 600 μL of DMEM medium with 10% FBS containing various concentrations of COS (0, 7, 14, and 21 mg/mL). After incubation for 24 h, cells on the upper surface of the membrane were gently removed with a cotton swab. The cells that invaded the lower surface of the membrane were washed with PBS and fixed with 4% POM for 20 min. After staining with crystal violet, the cells were photographed using a microscope.

### Flow cytometry assay

Following 48 treatments with MG63 and U2OS cells with COS (0, 7, 14, and 21 mg/mL), a flow cytometry assay was performed according to the manufacturer's protocol. The apoptosis rate was measured with FAC Scan Flow Cytometer (Becton–Dickinson, USA) followed by staining with AnnexinV-FITC/PI Apoptosis kit.

### Network pharmacology and molecular docking of COS

To investigate the inhibitory mechanism of COS on signaling pathways involved in regulating CEMIP expression, we conducted bioinformatics analyses, including prediction of potential target genes, construction of a protein–protein interaction (PPI) network, Kyoto Encyclopedia of Genes and Genomes (KEGG) pathway analysis, and molecular docking. The 3D chemical structural data of COS was obtained from PubChem (https://pubchem.ncbi.nlm.nih.gov/) [22–24] and then loaded into PharmMapper (http://www.lilab-ecust.cn/pharmmapper/) to predict potential target proteins through reverse screening [22, 24]. The PPI network was constructed using the STRING database (https://string-db.org/) to select target proteins arranged in order of fit score. Cytoscape (v3.9.1) was used to construct visualizations of the PPI network results and the interaction of targets. Human osteosarcoma-related gene data from the DisGeNET database (https:///www.disgenet.org/) was cross-matched with the Cytoscape analysis results. The obtained results were then uploaded to the KEGG database (https://www.kegg.jp/) for analysis and screening of signaling pathways and potential target proteins associated with COS. The 3D structure data of 6 target proteins (AKT1, EGFR, HRAS, HSP90AA1, PTK2, and IGF1R) were downloaded from the RCSB PDB database (http://www.rcsb.org/). Finally, Autodock and PyMOL were used to analyze and visualize COS-target protein docking.

### Quantitative real-time PCR (qRT-PCR) assay

TRIzol reagent (Invitrogen, CA, USA) was used to extract total RNA. The extracted RNA was reverse transcribed using the PrimeScript RT Reagent Kit (Takara Bio, Japan) according to the manufacturer’s instructions. Quantitative PCR was performed using the StepOnePlus Real-Time PCR system (Thermo Fisher Scientific). Thermocycling parameters were as follows: 95 °C for 20 s, 58 °C for 20 s, and 72 °C for 15 s, repeated for a total of 40 cycles. The primer sequences used in the present study were as follows: β-actin (human) forward CATGTACGTTGCTATCCAGGC and reverse CTCCTTAATGTCACGCACGAT; CEMIP (human) froward CACGGTCTATTCCATCCACATC and reverse GGTTCGCAAAACAATCGGCT. The expression of these genes was analyzed using the 2^–ΔΔCT^ method.

### Western blot analysis

After treating cells with COS for 48 h, total protein was extracted with radioimmunoprecipitation assay (RIPA) lysis buffer containing protease and phosphatase inhibitor (Solarbio, Beijing, China).

After determining protein concentration using the BCA Protein Assay Kit, the proteins were separated using Sodium Dodecyl Sulfate Polyacrylamide Gels. The proteins were blocked with 5% bovine serum albumin (BSA) for 1 h at room temperature, followed by transfer onto polyvinylidene difluoride membranes.

After incubating with primary antibodies at 4 °C overnight, the membranes were washed with TBS-T buffer solution for 30 min 3 times. The primary antibodies used were as follows: GAPDH (rabbit, 1:1000, Cat: 5174, CST), Bcl-2 (rabbit, 1:1000, Cat: AF6285, Beyotime), C-CAS3 (rabbit, 1:1000, Cat: AF1150, Beyotime), BAX (rabbit, 1:1000, Cat: AF0057, Beyotime), CEMIP (rabbit, 1:1000, Cat: A8587, ABclonal), AKT (rabbit, 1:1000, Cat: AF6261, Affinity), p-AKT (rabbit, 1:1000, Cat: AF3283, Affinity), PI3K (rabbit, 1:1000, Cat: AF7742, Beyotime), p-PI3K (rabbit, 1:1000, Cat: AF5905, Beyotime), mTOR (rabbit, 1:1000, Cat: AF6308, Affinity), and p-mTOR (rabbit, 1:1000, Cat: AF5869, Beyotime). After incubation with the primary antibodies, the membranes were incubated with a secondary antibody (1:2000, Cat: HA1001-100, Huabio) for 1 h at room temperature. The protein bands were visualized and analyzed with enhanced chemiluminescence (ECL Kit, Biosharp) and a Bio-Rad ChemiDoc system (Bio-Rad Laboratories, Inc.).

### Animal experiments

To confirm the inhibitory effect on CEMIP expression in vivo, we conducted animal experiments using Balb/C mice. The mice used in the in vivo experiments were purchased from the Shanghai Laboratory Animal Center of the Chinese Academy of Science and were acclimatized to predetermined conditions (12 h light/dark cycle, constant room temperature, and humidity) for 7 days.

K7M2 cells (5 × 10^6^cells) were inoculated subcutaneously in the right axillary region of the mice. After 6 days of inoculating osteosarcoma cells, the mice were randomly divided into two groups: negative control group (200 μL saline, intraperitoneal injection) and COS group (1 mg COS in 200 μL sterile PBS, intraperitoneal injection). Subsequently, intraperitoneal injections were given every 2 days for 22 days, after which the mice were euthanized, and the tumor tissues were removed for subsequent studies. Tumor volume were calculated as following formula: Tumor volume = length × (width) ^2^ /2.

### Immunohistochemistry

The expression of CEMIP and related pathway proteins in tumor tissue was examined using immunohistochemistry assays. The tumor samples were rehydrated in a graded series of ethanol (100%, 90%, 80%, 70%) and then deparaffinization with xylene. After blocking with 5% BSA, the slides were incubated with primary antibodies (CEMIP, p-AKT, p-mTOR, and p-PI3K) at 4 °C overnight. Then, the slides were stained with 3,3′-Diaminobenzidine tetrahydrochloride (DAB, BOSTER, China) and Hematoxylin, followed by incubation with secondary antibodies. The staining intensity and the percentage of stained cells were measured using Image J.

### Statistical analysis

SPSS 22.0 and Graph Pad Prism 8.0 were used for all statistical analyses. The data from the groups were compared using Analysis of Variance (ANOVA) and student’s t-tests. All results are expressed as mean ± standard deviation (SD). Each experiment was repeated 3 times independently. *P* < 0.05 was considered to indicate statistical significance (*P* < 0.05: *, *P* < 0.01: **, *P* < 0.001: ***, *P* < 0.0001: ****).

## Results

### COS inhibited the proliferation of osteosarcoma cells

**Fig. 1:**
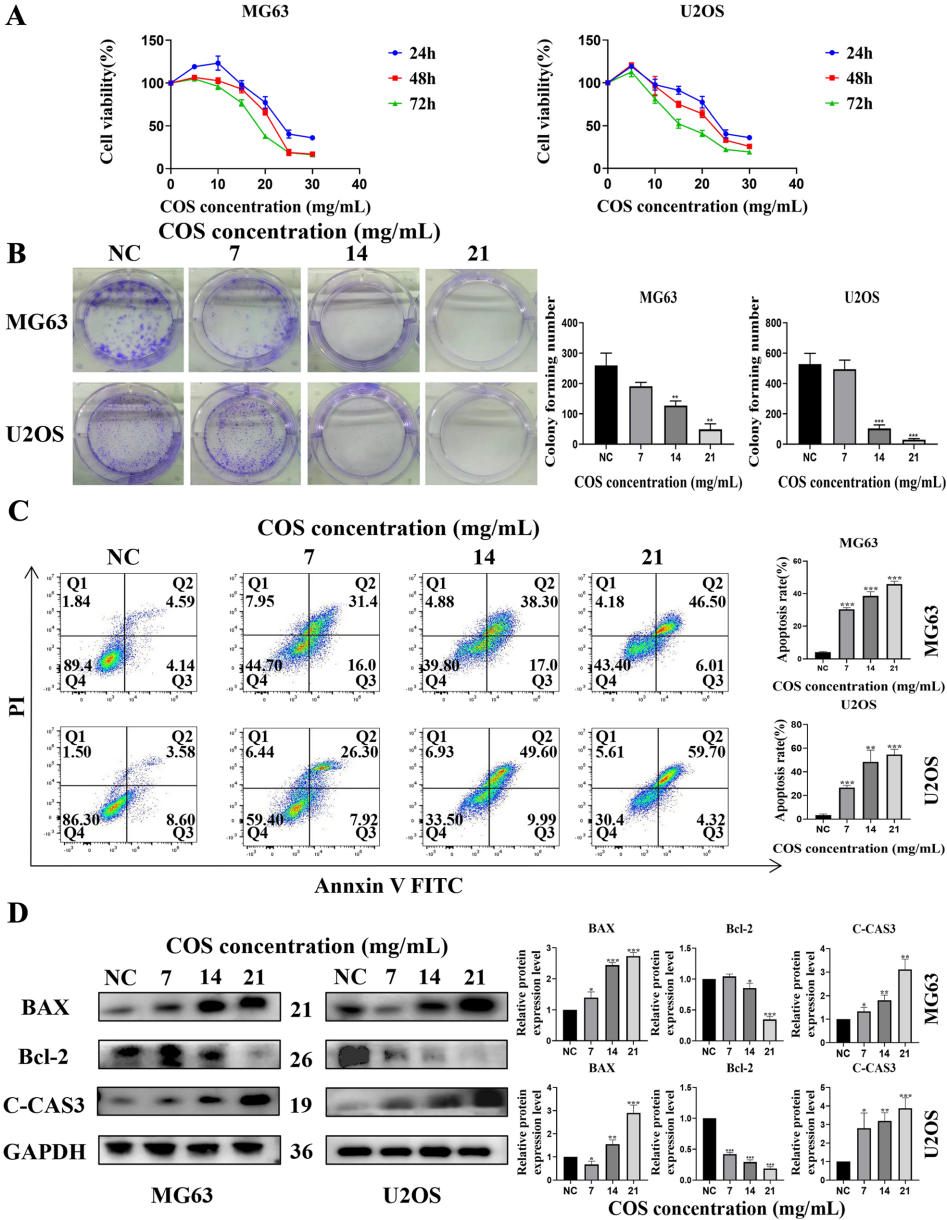
COS inhibited the proliferation of osteosarcoma cellsn **A** Human osteosarcoma cells (MG63 and U2OS) were treated with different concentrations of COS for 24 h, 48 h, and 72 h, respectively, and IC50 was calculated using Graph Pad Prism 8.0. **B** The colony formation of MG63 and U2OS cells was evaluated after COS treatment (0, 7, 14 and, 21 mg/mL). **C** Flow cytometry assay was performed to analyze apoptosis rates induced by COS treatment. Apoptosis rates in COS treatment groups were significantly higher than that in the NC group. **D** Western blot was conducted to evaluate pro-apoptotic proteins (BAX and C-Cas3) and anti-apoptotic protein (Bcl-2). All data are represented as mean ± SD; *p < 0.05, **p < 0.01, and ***p < 0.001. The experiments were repeated 3 times independently. Abbreviations: GAPDH, glyceraldehyde 3-phosphate dehydrogenase; Bcl-2, B-cell lymphoma-2; BAX, BCL2-Associated X; C-CAS3, cleaved caspase 3; NC, negative control; COS, Chitosan oligosaccharide

Cytotoxicity and inhibitory effects of COS on osteosarcoma cells were confirmed. The CCK-8 assay results showed that MG63 and U2OS cell viability was significantly inhibited by COS treatment (Fig. 1A); IC50 of COS in MG63 cells was 24.95 mg/mL (24 h), 21.58 mg/mL (48 h), 18.69 mg/mL (72 h), and IC50 in U2OS cells was 24.86 mg/mL (24 h), 21.84 mg/mL (48 h), 16.76 mg/mL (72 h). Thus, concentrations of 7 mg/mL (1/3 of IC50), 14 mg/mL (2/3 of IC50), and 21 mg/mL (IC50) were used in the subsequent experiments.

The results of the cell colony formation assay also confirmed the inhibitory effect of COS on the proliferation of osteosarcoma cells (Fig. 1B). Furthermore, flow cytometry was conducted to confirm the relationship between this inhibitory effect and apoptosis induction. Our results showed that COS significantly induced apoptosis in osteosarcoma cells (Fig. 1C). Moreover, we analyzed the expression of apoptosis-related proteins (BAX, Bcl-2, and C-Cas3), and the results showed that the expression of pro-apoptotic proteins (BAX and C-Cas3) was significantly increased in the COS treatment groups, while the expression of anti-apoptotic protein (Bcl-2) was decreased (Fig. 1D). These results suggest that COS exhibits an inhibitory effect on human osteosarcoma cells in vitro.

### COS suppressed the migration and invasion of osteosarcoma cells

**Fig. 2:**
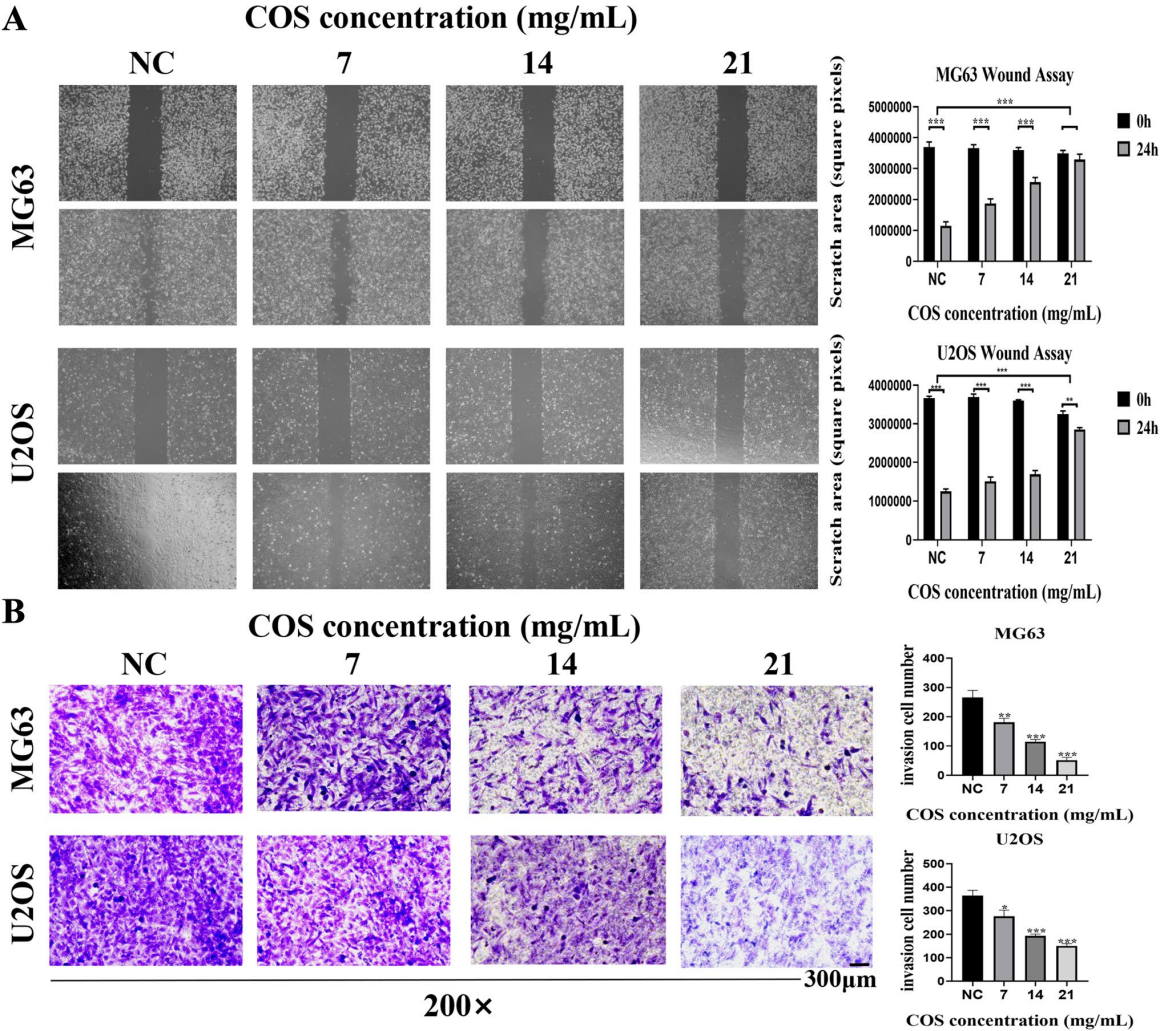
COS suppressed the migration and invasion of osteosarcoma cells. **A** Human osteosarcoma cells (MG63 and U2OS) were treated with various concentrations (0, 7, 14, and 21 mg/mL) of COS for 24 h, and migration ability was evaluated using wound healing assay. **B** Transwell cell invasion assay was performed after treating osteosarcoma cells with COS for 24 h. All data are represented as mean ± SD; *p < 0.05, **p < 0.01, and ***p < 0.001. The experiments were repeated 3 times independently. Abbreviations: NC, negative control; COS, Chitosan oligosaccharide

Further experiments were conducted to evaluate the effect of COS on the migration and invasion ability of osteosarcoma cells. The results of the wound healing assay demonstrated significant inhibition of osteosarcoma cell migration in the presence of COS (Fig. 2A). Similarly, in the transwell cell invasion test, COS also effectively inhibited the invasion of osteosarcoma cells in a dose-dependent manner (Fig. 2B). These findings demonstrate that COS could effectively inhibit the proliferation and metastasis of osteosarcoma cells in vitro.

### COS inhibited the expression of CEMIP in osteosarcoma cells

**Fig. 3:**
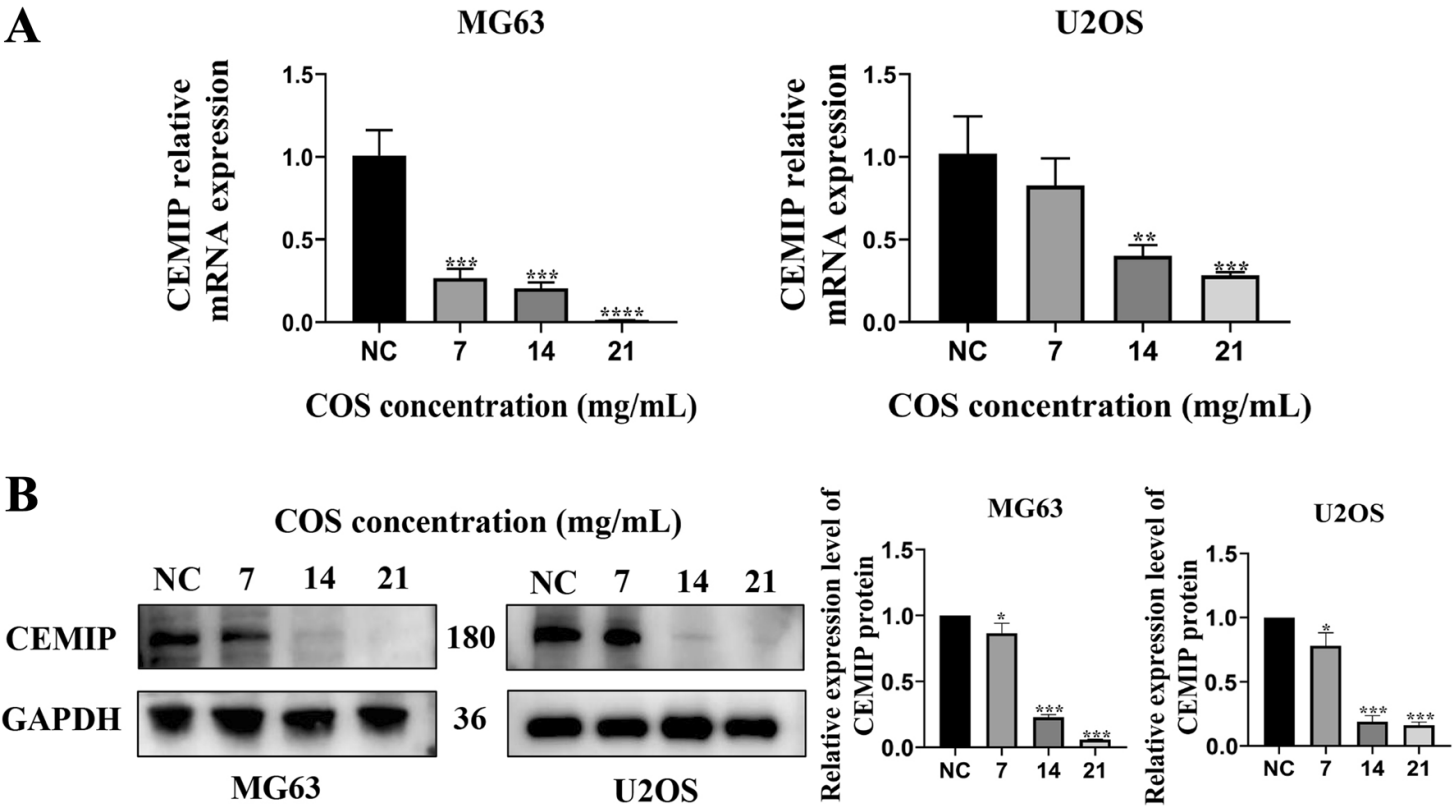
COS inhibited the expression of CEMIP in osteosarcoma cells. **A** MG63 and U2OS cells were treated with various doses (0, 7, 14 and, 21 mg/mL) of COS for 48 h, and mRNA expression level of CEMIP was evaluated with qRT-PCR. **B** The expression level of CEMIP protein of osteosarcoma cells was evaluated using Western blot followed by COS treatment. All data is expressed as mean ± SD; *p < 0.05, **p < 0.01, and ***p < 0.001. The experiments were repeated 3 times independently. Abbreviations: GAPDH, glyceraldehyde 3-phosphate dehydrogenase; CEMIP, Cell migration inducing protein; NC, negative control; COS, Chitosan oligosaccharide

Based on the observed inhibitory effect of COS on osteosarcoma cells, we investigated whether COS could affect the expression of CEMIP in osteosarcoma cells. Our findings revealed significant inhibition of CEMIP mRNA expression after COS treatment (Fig. 3A). Similarly, at the protein level, CEMIP expression was also inhibited in a dose-dependent manner (Fig. 3B). These results further support the findings that COS modulates CEMIP expression in osteosarcoma cells. Therefore, we conducted bioinformatics analysis to further explore the interrelationships between COS and CEMIP expression-related signaling pathways.

### Bioinformatics prediction for possible CEMIP expression-related signaling pathways of COS in osteosarcoma

**Fig. 4:**
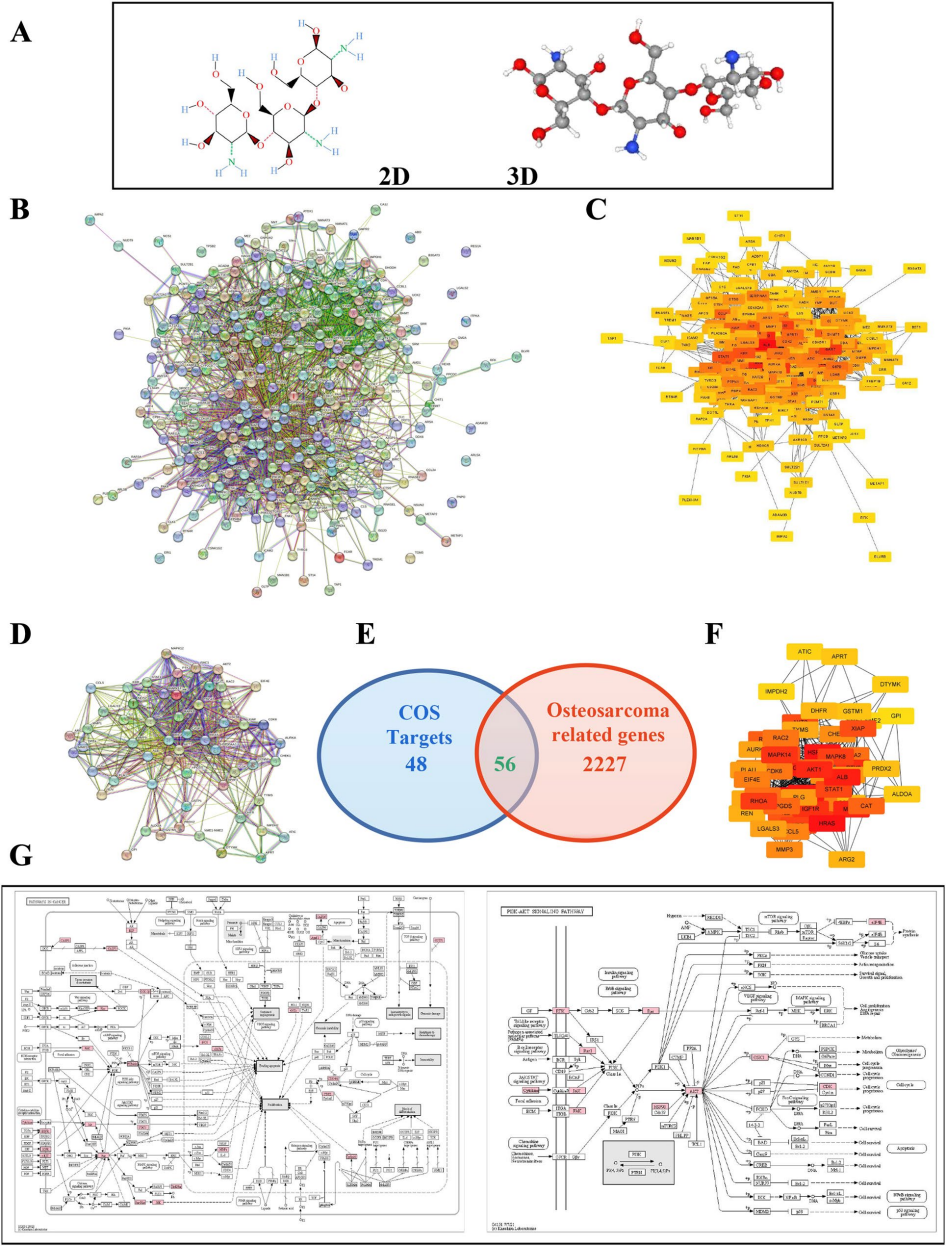
Bioinformatics prediction for possible target signaling pathways of COS in osteosarcoma. **A** 2D and 3D structure of COS. **B** A PPI network for possible target genes of COS was constructed to obtain possible edges by using STRING database. **C** 104 possible target genes were screened using Cytoscape software. **D**–**F** The intersection between COS target genes and human osteosarcoma-related genes was analyzed. **G** The primary associated pathways were screened from a key network of COS by KEGG pathway analysis. Abbreviations: PPI, protein–protein Interaction; KEGG, Kyoto Encyclopedia of Genes and Genomes; COS, Chitosan oligosaccharide

**Fig. 5:**
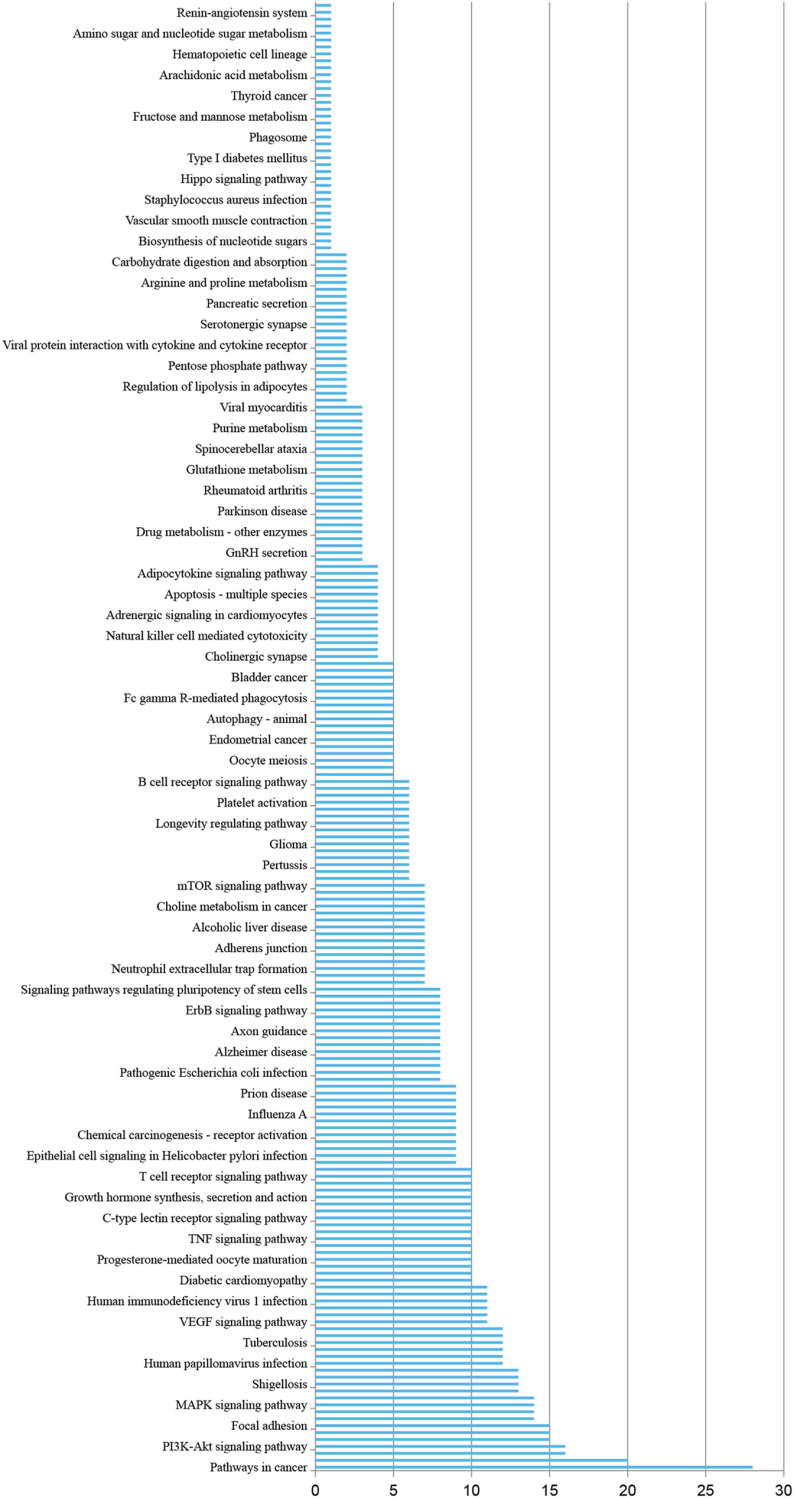
Possible target diseases, biological processes, and signaling pathways of COS. Diseases, biological processes, and signaling pathways related to COS target genes were screened using KEGG database analysis. Abbreviations: KEGG, Kyoto Encyclopedia of Genes and Genomes; COS, Chitosan oligosaccharide

**Fig. 6:**
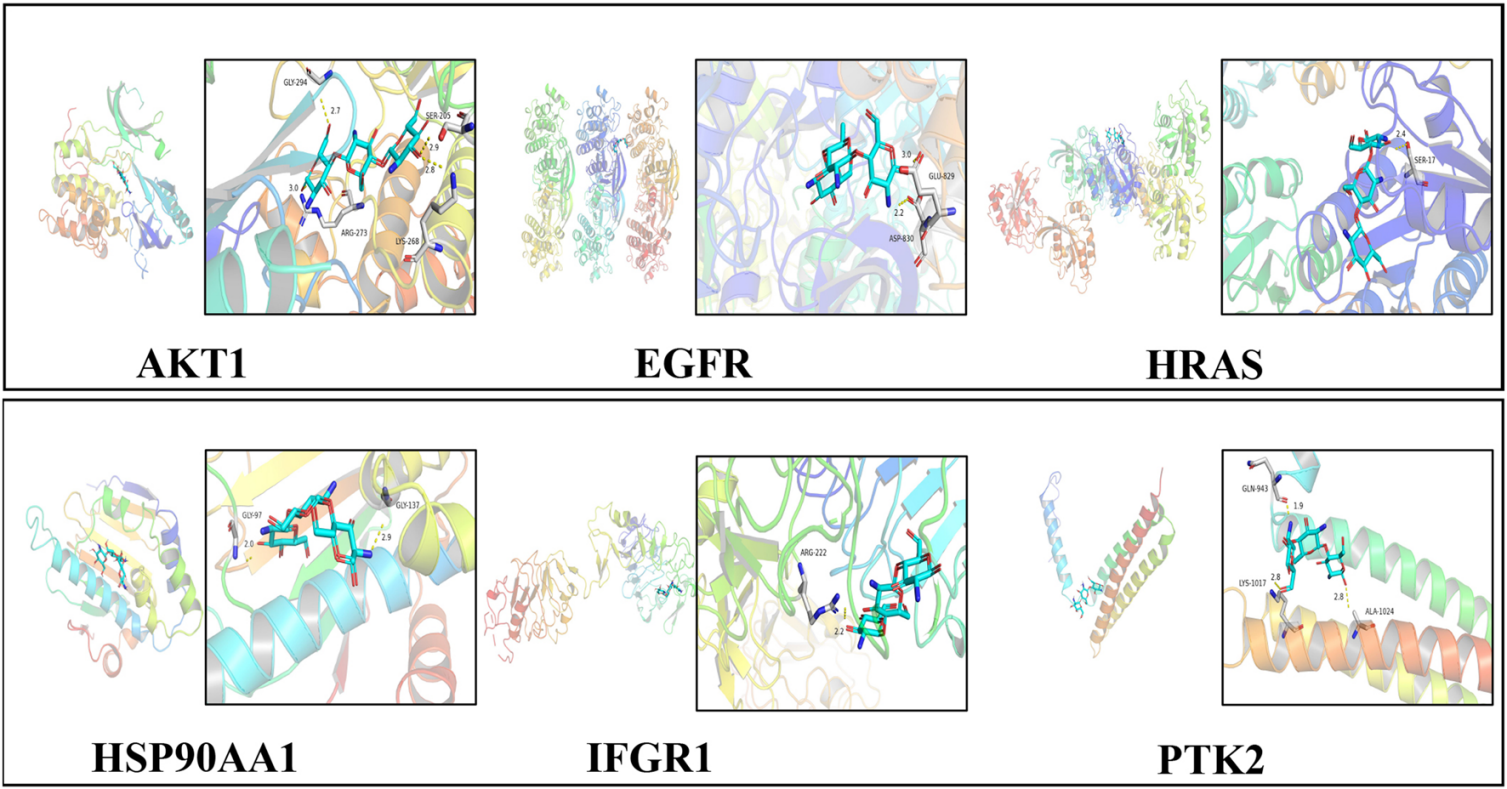
Docking of COS. The main target proteins (AKT1, EGFR, HRAS, HSP90AA1, IGFR1, and PTK2) were docked with COS. Abbreviations: AKT1, RAC-alpha serine/threonine-protein kinase; EGFR, Epidermal growth factor receptor; HRAS, GTPase HRas; HSP90AA1, Heat shock protein HSP 90-alpha; IGFR1, Insulin-like growth factor 1 receptor; PTK2, Protein-tyrosine kinase 2; COS, Chitosan oligosaccharide

In the bioinformatics analysis, 299 potential target genes of COS were identified using PharmMapper and arranged in fit score order. A PPI network was constructed for these target genes, and 2161 possible edges were obtained using the STRING database (Fig. 4B). Through further analysis using Cytoscape, 104 possible target genes with a degree score higher than the average (> 15.948) were selected (Fig. 4C). To establish their relevance in osteosarcoma, the 104 obtained COS target genes were compared with 2283 osteosarcoma-related genes obtained from the DisGeNET database (Fig. 4D–F). Relationship networks between 56 target points (Table 1) identified from the above analysis were constructed using the STRING database and Cytoscape. A total of 59 related genes were annotated using the UniProt database, and their functions and related signaling pathways were analyzed using the KEGG database. The KEGG analysis results showed that these target genes were involved in 212 diseases, biological processes, and signaling pathways, with 28 pathways being directly associated with cancer (13.21%), and 16 (7.55%) being involved in the PI3K signaling pathway (Fig. 4G and Fig. 5). From this analysis, it was found that many target signaling pathways of COS are related to cancer occurrence, with the PI3K signaling pathway having the highest proportion, suggesting that the PI3K signaling pathway may be the primary target of COS among several important cancer-related signaling pathways (Fig. 4G). Through KEGG and Cytoscape analysis, six key proteins (AKT1, EGFR, HRAS, HSP90AA1, IGFR1, and PTK2) were identified in the network. The 3D structure data of the key proteins were obtained from the PDB database, and the candidate target proteins were preprocessed and docked with COS molecules (Fig. 4A) using Autodock vina (Fig. 6).

**Table 1:** The list of 56 intersection genes with COS and osteosarcoma

Gene name	Uniprot ID	Protein name
EGFR	P00533	Epidermal growth factor receptor
AKT1	P31749	RAC-alpha serine/threonine-protein kinase
GSTP1	P09211	Glutathione S-transferase P
DHFR	P00374	Dihydrofolate reductase
PRDX2	P32119	Peroxiredoxin-2
MAPK14	Q16539	Mitogen-activated protein kinase 14
GSK3B	P49841	Glycogen synthase kinase-3 beta
IGF1R	P08069	Insulin-like growth factor 1 receptor
MMP9	P14780	Matrix metalloproteinase-9
PTK2	Q05397	Protein-tyrosine kinase 2
CASP3	P42574	Caspase-3
HPGDS	O60760	Hematopoietic prostaglandin D synthase
KIT	P10721	Mast/stem cell growth factor receptor Kit
MAPK8	P45983	Mitogen-activated protein kinase 8
ALB	P02768	Albumin
HSP90AA1	P07900	Heat shock protein HSP 90-alpha
RAC1	P63000	Ras-related C3 botulinum toxin substrate 1
CDK6	Q00534	Cyclin-dependent kinase 6
CHEK1	O14757	Serine/threonine-protein kinase Chk1
GSTM1	P09488	Glutathione S-transferase Mu 1
XIAP	P98170	E3 ubiquitin-protein ligase XIAP
MMP3	P08254	Matrix metalloproteinase-3
EIF4E	P06730	Eukaryotic translation initiation factor 4E
AKT2	P31751	RAC-beta serine/threonine-protein kinase
HRAS	P01112	GTPase HRas
JAK2	O60674	Tyrosine-protein kinase JAK2
KDR	P35968	Vascular endothelial growth factor receptor 2
MMP1	P03956	Matrix metalloproteinase-1
SRC	P12931	Proto-oncogene tyrosine-protein kinase Src
CDK2	P24941	Cyclin-dependent kinase 2
APRT	P07741	Adenine phosphoribosyltransferase
IL2	P60568	Interleukin-2
RHOA	P61586	Transforming protein RhoA
RAC2	P15153	Ras-related C3 botulinum toxin substrate 2
AURKA	O14965	Aurora kinase A
GPI	P06744	Glucose-6-phosphate isomerase
IMPDH2	P12268	Inosine-5'-monophosphate dehydrogenase 2
LGALS3	P17931	Galectin-3
ATIC	P31939	Bifunctional purine biosynthesis protein ATIC
PLAU	P00749	Urokinase-type plasminogen activator
CCL5	P13501	C–C motif chemokine 5
TYMS	P04818	Thymidylate synthase
CAT	P04040	Catalase
CCNA2	P20248	Cyclin-A2
DPP4	P27487	Dipeptidyl peptidase 4
DTYMK	P23919	Thymidylate kinase
ALDOA	P04075	Fructose-bisphosphate aldolase A
APAF1	O14727	Apoptotic protease-activating factor 1
ARG2	P78540	Arginase-2
NOS2	P35228	Nitric oxide synthase(NOS type II)
PLG	P00747	Plasminogen
REN	P00797	Renin
MAPK12	P53778	Mitogen-activated protein kinase 12
NOS2	P60321	Nanos homolog 2
STAT1	P42224	Signal transducer and activator of transcription 1-alpha/beta
CDC42	P60953	Cell division control protein 42 homolog

Based on the bioinformatics analysis, we predicted that COS has a strong regulatory effect on six major signaling pathway-related proteins (AKT1, EGFR, HRAS, HSP90AA1, IGFR1, and PTK2), having the highest affinity for the PI3K signaling pathway-related protein AKT1 (Table 2). Previous studies have also reported that CEMIP expression was influenced by the PI3K signaling pathway[20, 21].

**Table 2:** Affinity between six target proteins and COS

Gene name	Protein name	Affinity(kcal/mol)
AKT1	RAC-alpha serine/threonine-protein kinase	− 8.3
HRAS	GTPase HRas	− 8.1
IGFR1	Insulin-like growth factor 1 receptor	− 7.4
HSP90AA1	Heat shock protein HSP 90-alpha	− 6.8
EGFR	Epidermal growth factor receptor	− 6.2
PTK2	Protein-tyrosine kinase 2	− 4.9

Therefore, we hypothesize that the PI3K signaling pathway may be closely related to CEMIP expression, and the inhibitory effect of COS on CEMIP expression may also be associated with the regulation of PI3K signaling pathway activity. To validate this theory, we further investigated the impact of COS on PI3K signaling pathway activity.

### COS inhibited CEMIP expression via suppressing of PI3K/AKT/mTOR pathway in osteosarcoma cells

**Fig. 7:**
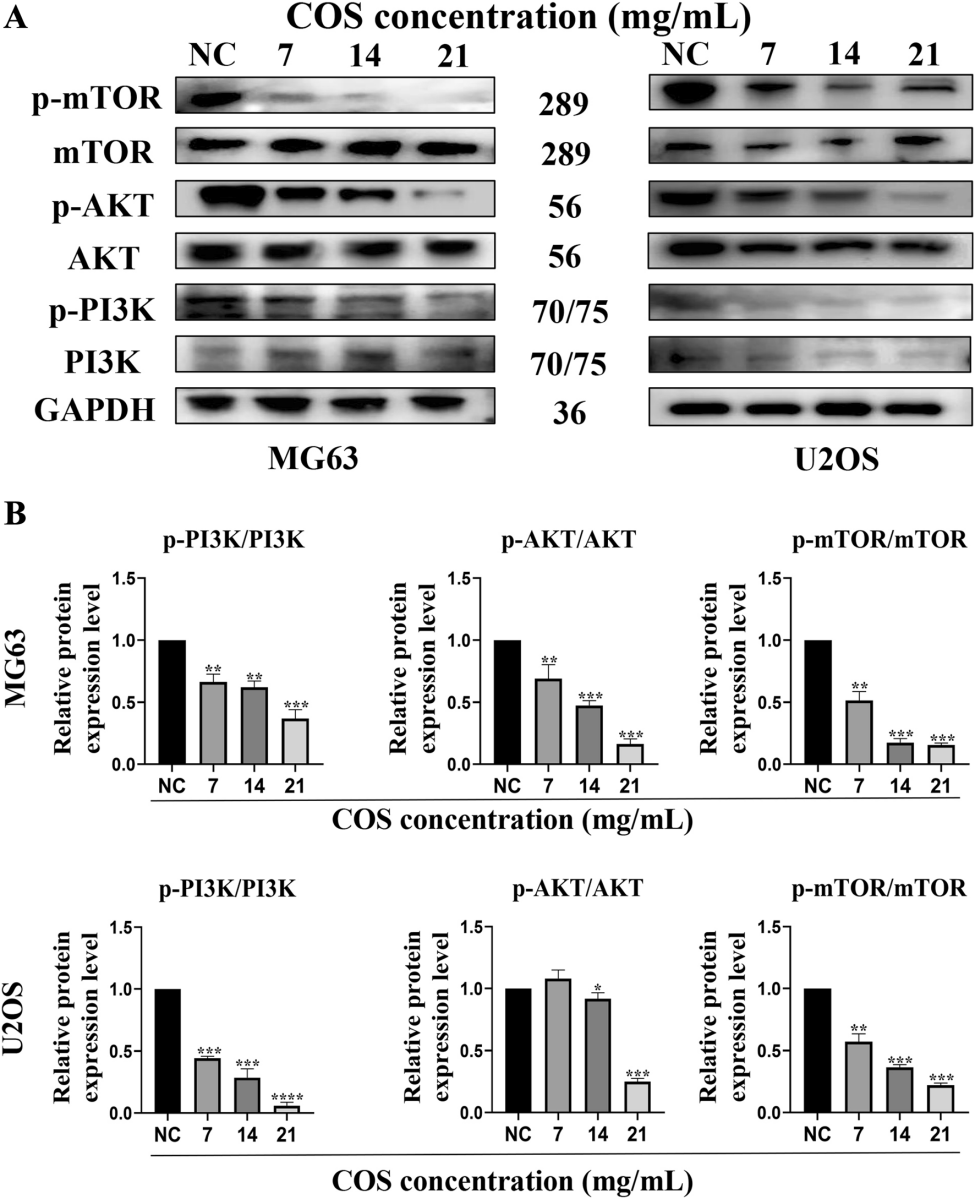
CEMIP expression was inhibited by COS via suppression of the PI3K/AKT/mTOR pathway in osteosarcoma cells. Western blot **A** and relevant quantitative analysis **B** of p-PI3K, p-AKT, and p-mTOR were performed after treating osteosarcoma cells with COS (0, 7, 14, and 21 mg/mL) for 48 h. All data are represented as mean ± SD; *p < 0.05, **p < 0.01, **p < 0.001, and ****p < 0.0001. The experiments were repeated 3 times independently. Abbreviations: GAPDH, glyceraldehyde 3-phosphate dehydrogenase; p-PI3K, phosphorylated phosphatidylinositol 3-kinase; p-AKT, phosphorylated RAC-alpha serine/threonine-protein kinase; p-mTOR, phosphorylated mammalian target of rapamycin; NC, negative control; COS, Chitosan oligosaccharide

Osteosarcoma cells (MG63 and U2OS) were seeded and cultured in 6-well plates and treated with various concentrations of COS for 48 h. The proteins in question were isolated, and their expression was analyzed at specified time points. Western Blot analysis revealed that when MG63 cells were treated with COS, the expression of p-PI3K, p-AKT, and p-mTOR was significantly reduced (Fig. 7A and B). The PI3K/AKT/mTOR pathway activity of U2OS cells was also significantly inhibited by COS treatment (Fig. 7A and B). These results indicated that COS could indeed interfere with the expression of CEMIP by inhibiting the PI3K/AKT/mTOR pathway.

### COS suppressed the progression of osteosarcoma and inhibited CEMIP expression in vivo

**Fig. 8:**
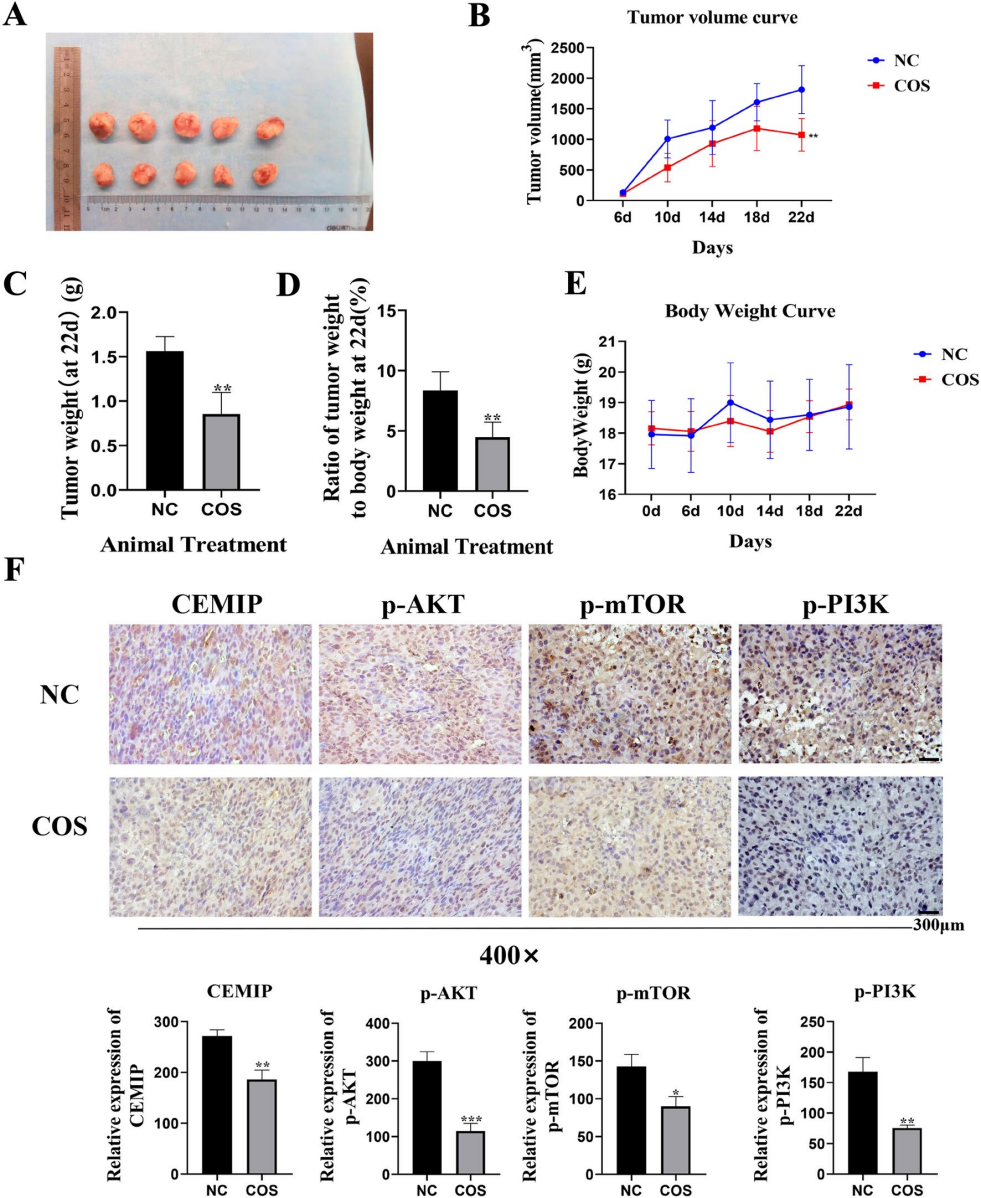
COS inhibited the progression of osteosarcoma and the expression of CEMIP in vivo. **A**–**E** Tumor size, volume, weight, body weight and ratio of tumor weight to body weight of mice were measured respectively. **F** Immunohistochemistry assay was performed to evaluate the expression of CEMIP and related pathway proteins in tumor tissue. All data are represented as mean ± SD; *p < 0.05, **p < 0.01, and ***p < 0.001. The experiments were repeated 3 times independently. Abbreviations: p-PI3K, phosphorylated phosphatidylinositol 3-kinase; p-AKT, phosphorylated RAC-alpha serine/threonine-protein kinase; p-mTOR, phosphorylated mammalian target of rapamycin; CEMIP, Cell migration inducing protein; NC, negative control; COS, Chitosan oligosaccharide

To confirm whether COS also has the same effect in vivo, we inoculated mice with osteosarcoma cells and treated them with COS. Treatment with COS significantly inhibited osteosarcoma growth. The tumors’ size, volume, weight, and the ratio of tumor weight to body weight were notably lower in the COS treatment group compared to the control group (Fig. 8A–D). There was no significant difference in body weight between the COS treatment group and the control group (Fig. 8E). We further analyzed the expression of CEMIP in tumor tissue with IHC assay and found that the expression of CEMIP in the COS-treated group was significantly lower than in the control group. Additionally, the expression of related signaling pathway proteins was significantly reduced in the COS-treated group compared to the control group (Fig. 8F). These results indicate that, like the effects observed in in vitro experiments, COS can also inhibit CEMIP expression in vivo.

## Discussion

Osteosarcoma is a malignant bone tumor that is prone to early metastasis and continues to have an overall survival rate with no significant improvement over the years [2]. Surgical therapy, chemotherapy, radiation therapy and other therapies are widely used to treat osteosarcoma, but approximately 30% of local osteosarcoma patients experience recurrence [25]. Consequently, active research endeavors explore methods and strategies that may suppress the malignancy of osteosarcoma and improve treatment efficacy. As the efforts to suppress osteosarcoma malignancy intensifies, the various factors related to osteosarcoma malignancy has started to become clearer. Research indicates that CEMIP is frequently overexpressed in various tumors and is closely related to poor prognosis [26–28]. Similarly, osteosarcoma patients with overexpressed CEMIP have a very poor prognosis [11]. Previous studies have demonstrated that CEMIP overexpression promotes tumor cell proliferation and metastasis, leading to enhanced tumor malignancy [3, 9, 20]. Several important signaling pathways, including the PI3K and Notch signaling pathways, have been implicated in the regulation of CEMIP expression [20, 21, 29]. Given the significant role that CEMIP plays in tumor malignancy, it has attracted considerable attention as a promising target for tumor therapy. Although there has been extensive research on the relationship between the overexpression of CEMIP and tumor malignancy, reports on the use of natural active compounds to inhibit its expression are currently limited.

COS is an oligomer isolated from chitin and exhibits good biological activity. This compound has been effectively used in many fields, including medicine, where it is recognized as an effective immune activator and a potential supplement for anti-tumor drugs [12, 13]. The inhibition of signaling pathway activity has been identified as one of the important mechanisms in the anti-tumor activity of COS [14, 30]. However, few reports have been done on the analysis of the specific signaling pathway proteins that interact with COS from a bioinformatics perspective.

The available data indicate that COS, as a naturally active compound with strong inhibitory activity against signaling pathways, may affect the expression of CEMIP. Therefore, based on the preliminary evidence of COS’s anti-tumor activity in osteosarcoma, we investigated the effect of COS on CEMIP expression.

First, we confirmed the inhibitory activity of COS on human osteosarcoma cells. Our results showed that COS significantly inhibited the proliferation and invasion of human osteosarcoma cells (MG63 and U2OS) and effectively induced apoptosis in vitro (Fig. 1–2). Based on this, we continued to investigate the effect of COS on CEMIP expression and found that COS significantly inhibited the expression of CEMIP in osteosarcoma at both the mRNA and protein levels.

The mechanism by which COS inhibited CEMIP expression was further explored through bioinformatics analysis to predict the relationship between CEMIP expression-related signaling pathways and COS. Although previous studies have reported that COS affects various signaling pathways, its precise target point has yet to be identified using bioinformatics analysis. Therefore, we dedicated considerable effort to this analysis. Bioinformatics is widely recognized as an effective research method for predicting accurate target points and is used to study interactions between small molecule compounds and signaling pathway targets [31–33]. In this study, bioinformatics analysis was conducted to predict the key signaling pathway target of COS.

104 possible target genes for COS were screened using PharmaMapper, STRING database, and Cytoscape. These genes were cross analyzed with human osteosarcoma-related genes (2283), and 56 potential target genes were obtained. Further analysis using the UniProt and KEGG databases revealed that COS exerts the greatest impact on proteins of the cancer-related PI3K signaling pathway. 6 key target proteins with the highest affinity were screened through docking with COS, among which AKT1 had the highest matching score (− 8.2), indicating that is COS most likely to interact with proteins of the PI3K signaling pathway (Table 2). Previous studies have also reported that the PI3K signaling pathway is closely related to the expression of CEMIP in various cancers[20, 21]. Therefore, based on the abovementioned bioinformatics predictions, further investigations were conducted to confirm whether COS indeed affects the expression of CEMIP by interfering with the PI3K/AKT/mTOR signaling pathway. Our results demonstrated that COS significantly inhibited the PI3K/AKT/mTOR signaling pathway, with subsequent effective inhibition of CEMIP expression. These results indicate that COS could significantly inhibit CEMIP expression in vitro. To validate these effects in vivo, animal experiments were conducted, and the results showed that the expression of CEMIP in the COS treatment group was significantly lower than that in the control group, with the expression of PI3K/AKT/mTOR signaling pathway protein also being significantly reduced. This result suggests that COS could also inhibit CEMIP expression in vivo.

The findings of our study collectively indicate that COS, a naturally active compound, may be effective in inhibiting the expression of CEMIP, one of the main factors associated with osteosarcoma malignancy.

The relationship between CEMIP expression and tumor malignancy has gained significant attention, and notable results have been obtained from research on its expression regulation[34–38]. The naturally active compound possesses modulatory properties on signaling pathways involved in various biological activities, making it a potential candidate for application, particularly in the medical field, as an effective adjunctive therapeutic agent in the treatment of tumors[39–44]. Further research is needed to explore the utilization of natural active compounds as complementary therapies for the treatment of osteosarcoma in the future.

## Conclusion

This study provides evidence that COS could inhibit the expression of CEMIP in osteosarcoma, which is closely associated with tumor malignancy. The inhibitory effect of COS may involve the inhibition of the PI3K/AKT/mTOR signaling pathway, as demonstrated with in vitro and in vivo experiments. These findings present new perspectives and offer a novel approach to osteosarcoma treatment from different perspectives.

## Data Availability

All datasets used or analyzed during the current study are available from the corresponding author on reasonable request.

## References

[1] He J, Zhang W, Di T, Meng J, Qi Y, Li G, Zhang Y, Su H, Yan W (2020). Water extract of sporoderm-broken spores of Ganoderma lucidum enhanced pd-l1 antibody efficiency through downregulation and relieved complications of pd-l1 monoclonal antibody. Biomed Pharmacother.

[2] Luetke A, Meyers PA, Lewis I, Juergens H (2014). Osteosarcoma treatment—where do we stand? A state of the art review. Cancer Treat Rev.

[3] Li L, Yan LH, Manoj S, Li Y, Lu L (2017). Central role of CEMIP in tumorigenesis and its potential as therapeutic target. J Cancer.

[4] Raish M, Khurshid M, Ansari MA, Chaturvedi PK, Bae SM (2012). Analysis of molecular cytogenetic alterations in uterine leiomyosarcoma by array-based comparative genomic hybridization. J Cancer Res Clin Oncol.

[5] Abe S, Usami S, Nakamura Y (2003). Mutations in the gene encoding KIAA1199 protein, an inner-ear protein expressed in Deiters’ cells and the fibrocytes, as the cause of nonsyndromic hearing loss. J Hum Genet.

[6] Kohi S, Sato N, Koga A, Matoyoshi N, Hirata K (2017). KIAA1199 is induced by inflammation and enhances malignant phenotype in pancreatic cancer. Oncotarget.

[7] Zhang D, Zhao L, Shen Q, Lv Q, Jin M, Ma H, Nie X, Zheng X, Huang S, Zhou P, Wu G, Zhang T (2017). Down-regulation of KIAA1199/CEMIP by miR-216a suppresses tumor invasion and metastasis in colorectal cancer. Int J Cancer.

[8] Michishita E, Garcés G, Barret JC, Horikawa I (2006). Upregulation of the KIAA1199 gene is associated with cellular mortality. Cancer Lett.

[9] Jami M, Hou J, Liu M, Varney ML, Hassan H, Dong J, Geng L, Wang J, Yu F, Huang X, Peng H, Fu K, Li Y, Singh RK, Ding SJ (2014). Functional proteomic analysis reveals the involvement of KIAA1199 in breast cancer growth, motility and invasiveness. BMC Cancer.

[10] Koga A, Sato N, Kohi S, Yabuki K, Cheng XB, Hisaoka M, Hirata K (2017). KIAA1199/CEMIP/HYBID overexpression predicts poor prognosis in pancreatic ductal adenocarcinoma. Pancreatology.

[11] Ito K, Nishida Y, Ikuta K, Urakawa H, Koike H, Sakai T, Zhang J, Shimoyama Y, Imagama S (2021). Overexpression of KIAA1199, a novel strong hyaluronidase, is a poor prognostic factor in patients with osteosarcoma. J Orthop Surg Res.

[12] Muanprasat C, Chatsudthipong V (2017). Chitosan oligosaccharide: biological activities and potential therapeutic applications. Pharmacol Ther.

[13] Aam BB, Heggset EB, Norberg AL, Sorlie M, Varum KM, Eijsink VGH (2010). Production of chitooligosaccharides and their potential applications in medicine. Mar Drugs.

[14] Luo ZG, Dong XX, Ke Q, Duan QW, Shen L (2014). Downregulation of CD147 by chitooligosaccharide inhibits MMP-2 expression and suppresses the metastatic potential of human gastric cancer. Oncol Lett.

[15] Jing B, Cheng G, Li J, Wang ZA, Du Y (2019). Inhibition of liver tumor cell metastasis by partially acetylated chitosan oligosaccharide on a tumor-vessel microsystem. Mar Drugs.

[16] Mei YX, Chen HX, Zhang J, Zhang XD, Liang YX (2013). Protective effect of chitooligosaccharides against cyclophosphamide-induced immunosuppression in mice. Int J Biol Macromol.

[17] Zhang G, Cheng G, Jia P, Jiao S, Feng C, Hu T, Liu H, Du Y (2017). The positive correlation of the enhanced immune response to PCV2 subunit vaccine by conjugation of chitosan oligosaccharide with the deacetylation degree. Mar Drugs.

[18] Mayer IA, Arteaga CL (2016). The PI3K/AKT pathway as a target for cancer treatment. Annu Rev Med.

[19] Comprehensive molecular portraits of human breast tumours (2012). Nature.

[20] Shen F, Zong ZH, Liu Y, Chen S, Sheng XJ, Zhao Y (2019). CEMIP promotes ovarian cancer development and progression via the PI3K/AKT signaling pathway. Biomed Pharmacother.

[21] Mi C, Zhang D, Li Y, Ren M, Ma W, Lu G, He S (2021). miR-4677-3p participates proliferation and metastases of gastric cancer cell via CEMIP-PI3K/AKT signaling pathway. Cell Cycle.

[22] Wang X, Shen Y, Wang S, Li S, Zhang W, Liu X, Lai L, Pei J, Li H (2017). PharmMapper 2017 update: a web server for potential drug target identification with a comprehensive target pharmacophore database. Nucleic Acids Res.

[23] Jiang Y, Zhang M, Wang L, Zhang L, Ma M, Jing M, Li J, Song R, Zhang Y, Yang Z, Zhang Y, Pu Y, Qu X, Fan J (2023). Potential mechanisms of osthole against bladder cancer cells based on network pharmacology, molecular docking, and experimental validation. BMC Complement Med Ther.

[24] He J, Zhang W, Zhou X, Yan W, Wang Z (2021). Aloin induced apoptosis by enhancing autophagic flux through the PI3K/AKT axis in osteosarcoma. Chin Med.

[25] Pilavaki P, Ardakani AG, Gikas P, Constantinidou A (2023). Osteosarcoma: current concepts and evolutions in management principles. J Clin Med.

[26] Shen X, Mo X, Tan W, Mo X, Li L, Yu F, He J, Deng Z, Xing S, Chen Z, Yang J (2022). KIAA1199 correlates with tumor microenvironment and immune infiltration in lung adenocarcinoma as a potential prognostic biomarker. Pathol Oncol Res.

[27] Domanegg K, Sleeman JP, Schmaus A (2022). CEMIP, a promising biomarker that promotes the progression and metastasis of colorectal and other types of cancer. Cancers.

[28] Dong X, Yang Y, Yuan Q, Hou J, Wu G (2021). High expression of CEMIP correlates poor prognosis and the tumur microenvironment in breast cancer as a promisingly prognostic biomarker. Front Genet.

[29] Cheng J, Zhang Y, Wan R, Zhou J, Wu X, Fan Q, He J, Tan W, Deng Y (2022). CEMIP promotes osteosarcoma progression and metastasis through activating notch signaling pathway. Front Oncol.

[30] Pan Z, Cheng DD, Wei XJ, Li SJ, Guo H, Yang QC (2021). Chitooligosaccharides inhibit tumor progression and induce autophagy through the activation of the p53/mTOR pathway in osteosarcoma. Carbohydr Polym.

[31] Chen M, Jing Y, Wang L, Feng Z, Xie XQ (2019). DAKB-GPCRs: an integrated computational platform for drug abuse related GPCRs. J Chem Inf Model.

[32] Petta I, Lievens S, Libert C, Tavernier J, Bosscher KD (2016). Modulation of protein-protein interactions for the development of novel therapeutics. Mol Ther.

[33] Abyadeh M, Yadav VK, Kaya A (2023). Common molecular signatures between coronavirus infection and Alzheimer’s disease reveal targets for drug development. bioRxiv.

[34] Xu G, Zhao L, Hua Q, Wang L, Liu H, Lin Z, Jin M, Wang J, Zhou P, Yang K, Wu G, Yu D, Zhang D, Zhang T (2023). CEMIP, acting as a scaffold protein for bridging GRAF1 and MIB1, promotes colorectal cancer metastasis via activating CDC42/MAPK pathway. Cell Death Dis.

[35] Liu M, Xie L, Zhang Y, Chen J, Zhang X, Chen Y, Huang W, Cai M, Liang L, Lai M, Huang J, Guo Y, Lin L, Zhu K (2023). Inhibition of CEMIP potentiates the effect of sorafenib on metastatic hepatocellular carcinoma by reducing the stiffness of lung metastases. Cell Death Dis.

[36] Zhou M, Hua W, Sun Y (2022). Cell migration inducing hyaluronidase 1 promotes growth and metastasis of papillary thyroid carcinoma. Bioengineered.

[37] Liu B, Li X, Wang D, Yu Y, Lu D, Chen L, Lv F, Li Y, Cheng L, Song Y, Xing Y (2022). CEMIP promotes extracellular matrix-detached prostate cancer cell survival by inhibiting ferroptosis. Cancer Sci.

[38] Chen Y, Zhou H, Jiang WJ, Wang JF, Tian W, Jiang Y, Xia BR (2022). The role of CEMIP in tumors: An update based on cellular and molecular insights. Biomed Pharmacother.

[39] Mir RH, Mir PA, Uppal J, Chawla A, Patel M, Bardakci F, Adnan M, Mohi-ud-din R (2023). Evolution of natural product scaffolds as potential proteasome inhibitors in developing cancer therapeutics. Metabolites.

[40] Siddiqui AJ, Jaha S, Singh R, Saxena J, Asharaf SA, Khan A, Choudhary RK, Balakrishnan S, Badrauoi R, Bardakci F, Adnan M (2022). Plants in anticancer drug discovery: from molecular mechanism to chemoprevention. Biomed Res Int.

[41] Boueroy P, Hahnajanawong W, Boonmars T, Saensa-ard S, Anantachoke N, Vaeteewoottacharn K, Reutrakul V (2016). Antitumor effect of forbesione isolated from *Garcinia hanburyi* on cholangiocarcinoma in vitro and in vivo. Oncol Lett.

[42] Hu S, Zheng W, Jin L (2021). Astragaloside IV inhibits cell proliferation and metastasis of breast cancer via promoting the long noncoding RNA TRHDE-AS1. J Nat Med.

[43] Li W, Hu X, Li Y, Song K (2021). Cytotoxicity and growth-inhibiting activity of *Astragalus* polysaccharides against breast cancer via the regulation of EGFR and ANXA1. J Nat Med.

[44] Kitagawa T, Matsumoto T, Imahori D, Kobayashi M, Okayama M, Ohta T, Yoshida T, Watanabe T (2021). Limonoids isolated from the *Fortunella crassifolia* and the Citrus junos with their cell death-inducing activity on Adriamycin-treated cancer cell. J Nat Med.

